# Psychotropic medication use and bone loss in men: longitudinal study

**DOI:** 10.1192/bjo.2026.10985

**Published:** 2026-03-11

**Authors:** D. Kavindi Weerasinghe, Amanda L. Stuart, Julie A. Pasco, Mohammadreza Mohebbi, Jason M. Hodge, Rasika M. Samarasinghe, Lana J. Williams

**Affiliations:** Deakin University, School of Medicine, IMPACT, The Institute for Mental and Physical Health and Clinical Translation, Geelong, Australia; Barwon Health, University Hospital, Geelong, Australia; Department of Medicine-Western Health, The University of Melbourne, Australia; Biostatistics Unit, Faculty of Health, Deakin University, Geelong, Australia

**Keywords:** Psychotropic, antidepressants, antipsychotics, anticonvulsants, bone loss

## Abstract

**Background:**

Psychotropic medication use has been shown to be associated with decreased bone mineral density (BMD) and quality, and increased fracture risk. Less is known about psychotropic use and associated bone loss over time.

**Aims:**

To determine the association between psychotropic medication use and bone loss in men.

**Method:**

Data from 940 men (aged ≥20 years) participating in the Geelong Osteoporosis Study were used in this longitudinal study. BMD (g/cm^2^) at the spine and hip were measured with dual-energy X-ray absorptiometry at baseline, and 5 and 15 years post-baseline. Body mass index (BMI) was calculated, lifestyle factors and medication use was self-reported, and socioeconomic status was determined. Mood and anxiety disorders were identified through a clinical interview. Multivariable linear regression was used to determine the associations.

**Results:**

Over the study period (median 13.2 years), psychotropic use was associated with change in BMD at the spine (unadjusted mean difference −0.063 g/cm^2^, 95% CI −0.096 to −0.031, *p* < 0.001) and hip (−0.038 g/cm^2^, 95% CI −0.059 to −0.017, *p* < 0.001). BMI was identified as an effect modifier. Psychotropic use was associated with spine and hip bone loss at the 25th (adjusted mean difference −0.077g/cm^2^ (95% CI −0.122 to −0.033); and −0.058 g/cm^2^ (95% CI −0.084 to −0.032), respectively) and 50th percentile (adjusted mean difference −0.053 g/cm^2^ (95% CI −0.089 to −0.018) and −0.038 g/cm^2^ (95% CI −0.059 to −0.017), respectively), but not the 75th percentile of BMI (*p* = 0.121 and *p* = 0.106, respectively).

**Conclusions:**

Psychotropic use was associated with bone loss in non-obese men, highlighting the need for regular monitoring and preventive strategies to protect bone health.

Mental health is an important component of overall health and well-being, and is a major contributor to disability and morbidity.^1^ In Australia, one in five people aged 16−85 years experience a mental disorder at some point in their life.^1^ Psychotropic medications such as antidepressants, antipsychotics and anticonvulsants are used to treat these disorders, with more than one in six Australians (18%) filling a mental health-related prescription between 2021 and 2022 − a 3% increase from 2017 to 2018.^2^ Antidepressants were the most commonly prescribed, accounting for 74% of all mental health-related medications dispensed.^2^

There are concerns regarding the association between chronic psychotropic use and metabolic disturbances such as obesity, hypertension, diabetes, cardiovascular problems and changes in intestinal flora.^3^ Growing evidence also suggests that the use of certain psychotropic medications are associated with lower bone mineral density (BMD),^4–8^ poorer bone quality^9,10^ and increased risk of osteoporosis and subsequent fractures.^11,12^ Having a mental disorder has also been shown to coincide with poorer bone health independent of psychotropic medication use.^13,14^ According to the Australian Bureau of Statistics 2020–2021 National Health Survey, 27% of Australians are affected by a chronic musculoskeletal condition, with 3.4% having osteoporosis or osteopenia.^15^ A recent meta-analysis that pooled data from 70 eligible studies of women (*N* = 800 457) and 40 eligible studies of men (*N* = 453 964) reported that 23.1% of women and 11.7% of men had osteoporosis.^16^

To date, the majority of studies investigating the associations between psychotropic medication use and bone health are cross-sectional in nature, include women only or have investigated antidepressant use only, particularly selective serotonin reuptake inhibitors (SSRIs), with limited data on men. This gap has important public health implications, as it limits our ability to develop targeted prevention and management strategies for bone health in all populations. Therefore, this study aimed to investigate the effects of psychotropic medication use on bone loss over time in a population-based cohort of men spanning the full adult age range. Given previous findings suggesting psychotropic medication use is associated with decreased BMD, we hypothesised that psychotropic use exacerbates bone loss over time.

## Method

### Participants

This study utilised data from men participating in the Geelong Osteoporosis Study, a population-based, cohort study located in South-East Australia. Between 2001 and 2006, 2296 men were randomly selected from the electoral rolls for the Barwon Statistical Division, with 1540 agreeing to participate (67% response).^17^ Participants returned for follow-up assessments at 5 and 15 years post-baseline (*n* = 978, 81% response and *n* = 629, 72% response, respectively). Lithium users (*n* = 4) were removed because of its potential positive effects on BMD.^18^ Those with BMD measurements at two or more assessments were eligible for inclusion in the current study, resulting in a sample of 940 men. All study participants gave written informed consent to participate in the study, which was approved by the Barwon Health Human Research Ethics Committee (identifier 00/56).

### Measures

#### Outcome

Areal BMD (g/cm^2^) at the lumbar spine (L2–L4) and total hip was measured with dual-energy X-ray absorptiometry (DXA) at each assessment. Lunar DPX-L (software version 1.31; Lunar, Madison, Wisconsin, USA) was used for the first 544 men at baseline, and GE-Prodigy (Prodigy; GE Lunar, Madison, Wisconsin, USA) was used thereafter as the machine was updated.^17^ The two scanners were cross-calibrated before the DPX-L was decommissioned, and no significant differences in mean lumbar spine or femoral neck BMD were identified.^19^ An anthropomorphic phantom (Hologic) was scanned three times a week to monitor long-term stability of both devices.^19^

#### Exposures

Current medication use was documented at each assessment. To obtain accurate information, participants were asked to bring prescriptions or containers to their appointment. Use of antidepressants, antipsychotics and anticonvulsants were recorded, and use of any of the three was referred to as ‘any psychotropic use’ in this manuscript. Use of antidepressants was further categorised as SSRIs, serotonin norepinephrine reuptake inhibitors (SNRIs), tricyclic antidepressants (TCAs) and other (tetracyclic antidepressants, monoamine oxidase inhibitors and atypical antidepressants). Medications known to affect bone were categorised as either having a positive (anti-resorptive agents and gonadal hormones (testosterone)) or negative (oral glucocorticoids and thyroid hormone) effect. Date of initiation and dose were documented.

The Structured Clinical Interview for the DSM-IV Non-Patient Edition (SCID-I/NP)^20^ was used to identify a history of mood and anxiety disorders. SCID-I/NP data were available from the 5-year (2007–2011) and 15-year (2015–2019) time points. Age at onset was also determined.

Weight and height were measured with electronic scales and a Harpenden wall-mounted stadiometer. Body mass index (BMI) was calculated by using weight and height measures (kg/m^2^). Current smoking was recorded as smoker or non-smoker based on use of manufactured or hand-rolled cigarettes, cigars or pipes at the time of assessment. Daily alcohol usage was recorded via a food-frequency questionnaire, and analysed as grams per day.^21^ Mobility was recorded as active (move, walk, work energetically, does normal housework/other work, engage in light exercise) versus inactive (sedentary to bedfast) based on Metabolic Equivalent of Task Values, as previously described.^17^ The Index of Relative Socio-economic Disadvantage scores were used to indicate area-based socioeconomic status, where quintile 1 represents most disadvantaged and quintile 5 represents the least disadvantaged.^22^ These were determined from Socio-Economic Index for Areas index scores, using participants residential addresses based on the Australian Bureau of Statistics census data of income (low to high) and occupation (unskilled employment to professional positions).

### Statistical analyses

Differences in characteristics between psychotropic medication users and non-users at baseline were explored with chi-squared tests (Fisher’s exact test was used if required) for categorical variables and Kruskal–Wallis tests for non-parametric continuous variables. Linear regression techniques with robust Huber–White standard errors were used to estimate cluster-robust standard error, to address repeated outcome measurements in the longitudinal data.^23^ Regression models estimated the longitudinal association between psychotropic medication use and BMD over time (i.e. baseline, year 5 and 15 measurements) at the lumbar spine and total hip. Time-updated confounders included age, BMI, smoking, alcohol intake, physical activity level, use of medications known to affect bone, socioeconomic status and history of mood/anxiety disorders. Two-way interactions were checked in the final model and included if significant (*p* < 0.05). Unadjusted (model 1), age- and BMI-adjusted (model 2) and fully adjusted (model 3) models are presented. Statistical analyses were performed using Minitab (version 10.0.14393, build 14393 for Windows; State College, Pennsylvania, USA; https://www.minitab.com) and Stata 18 for Windows (StataCorp, College Station, Texas, USA; https://www.stata.com/stata18/).

## Results

### Baseline characteristics

At baseline, 59 men (59/940; 6.1%) were identified as using psychotropic medication. There were no differences detected between psychotropic medication users and non-users regarding age, BMI, alcohol intake, use of bone active medications, socioeconomic status, activity level or spine BMD. However, a higher proportion of psychotropic medication users were smokers and more likely to have a history of mental disorders than non-users (Table 1). Hip BMD was lower in psychotropic medication users compared with non-users. Participants were followed for a median of 13.2 years (interquartile range (IQR): 5.2–14.1), referred to as the study period hereafter.

**Table 1:** Baseline characteristics of the psychotropic users and non-users

Variables	Psychotropic user *n* = 59	Non-user *n* = 881	*p*-value
Age, years, median (IQR)	57.6 (43.1, 71.8)	53.9 (40.3, 67.7)	0.212
Body mass index, kg/m^2^, median (IQR)	26.7 (24.4, 30.6)	26.8 (24.4, 29.2)	0.357
Smoking, current, *n* (%)	15 (25.4)	124 (14.1)	0.017
Alcohol intake, g/day, median (IQR)	13.3 (0.7, 32.5)	14.3 (2.5, 29.9)	0.437
Physically active, *n* (%)	42 (71.2)	715 (81.2)	0.060
Medication use, current, *n* (%)			
Antiresorptive agents	0 (0.0)	7 (0.8)	1.000
Hormone replacement	0 (0.0)	5 (0.6)	1.000
Oral glucocorticoids	1 (1.7)	10 (1.1)	0.512
Thyroid hormones	1 (1.7)	4 (0.5)	0.277
Socioeconomic status, *n* (%)			
Quintile 1 (most disadvantaged)	10 (17.0)	144 (16.4)	
Quintile 2	13 (22.0)	164 (18.6)	
Quintile 3	6 (10.2)	170 (19.3)	0.168
Quintile 4	8 (13.6)	173 (19.6)	
Quintile 5 (least disadvantaged)	22 (37.3)	230 (26.1)	
Mood and/or anxiety disorder, lifetime, *n* (%)	32 (54.2)	123 (14.0)	<0.001
Bone mineral density (g/cm^2^), mean (s.d.)			
Spine	1.249 (0.228)	1.284 (0.189)	0.256
Total hip	0.964 (0.153)	1.010 (0.149)	0.029

Note: results are presented as *n* (%), mean (SD) or median (IQR).

IQR, interquartile range.

### Psychotropic medication use

One hundred and thirty-eight (14.7%) men used a psychotropic medication (antidepressants, antipsychotics, anticonvulsants) at one or more time points, with a median of 45 months (IQR 12.0–118.3). Psychotropic medication use was associated with reduced lumbar spine BMD over the study period (model 1, Table 2). Following adjustment for age and BMI (model 2) and further adjustment for smoking, alcohol intake, physical activity level, use of bone active medications, socioeconomic status and history of mood and anxiety disorders (model 3), the relationship remained (Table 2). In the final model (model 3), there was a significant interaction identified between psychotropic use and BMI. Psychotropic use was associated with reduced lumbar spine BMD over the study period at the 25th percentile of BMI (adjusted mean difference −0.077 g/cm^2^, 95% CI −0.122 to −0.033, *p* = 0.001) and the 50th percentile (adjusted mean difference −0.053 g/cm^2^, 95% CI −0.089 to −0.018, *p* = 0.003), but no relationship was detected at the 75th percentile (*p* = 0.121; Table 3), indicating the association was stronger among those with lower BMI.

**Table 2:** Unadjusted and adjusted linear regression models for psychotropic medication use and change in bone mineral density at the spine and total hip (g/cm^2^)

Variables	Model 1			Model 2			Model 3		
	Unadjusted mean difference	95% CI	*p*-value	Model-adjusted mean difference	95% CI	*p*-value	Model-adjusted mean difference	95% CI	*p*-value
Psychotropic use^a^									
Spine	−0.063	−0.096 to −0.031	<0.001	−0.070	−0.100 to −0.036	<0.001	−0.044	−0.081 to −0.008	0.016
Total hip	−0.038	−0.059 to −0.017	<0.001	−0.037	−0.056 to −0.018	<0.001	−0.031	−0.052 to −0.009	0.005
Antidepressant use									
Spine	−0.056	−0.091 to −0.021	0.002	−0.062	−0.097 to −0.027	<0.001	−0.032	−0.070 to 0.006	0.101
Total hip	−0.021	−0.044 to 0.002	0.077	−0.022	−0.043 to −0.001	0.042	−0.013	−0.038 to 0.011	0.289
Antipsychotic use									
Spine	−0.003	−0.138 to 0.131	0.962	−0.031	−0.164 to 0.103	0.651	−0.022	−0.109 to 0.066	0.629
Total hip	0.005	−0.082 to 0.091	0.916	−0.055	−0.133 to 0.023	0.165	−0.023	−0.110 to 0.063	0.596
Anticonvulsant use									
Spine	−0.081	−0.150 to −0.012	0.022	−0.075	−0.144 to −0.007	0.031	−0.067	−0.146 to 0.013	0.101
Total hip	−0.106	−0.151 to −0.062	<0.001	−0.080	−0.120 to −0.036	<0.001	−0.071	−0.110 to −0.031	<0.001

Note: model 1 is unadjusted; model 2 is adjusted for age and body mass index; model 3 is adjusted for age, body mass index, smoking, alcohol intake, physical activity level, use of bone positive and negative medications, Index of Relative Socio-economic Disadvantage score and any mood/anxiety disorder.

a. Antidepressant, antipsychotic and anticonvulsant users are included as psychotropic users.

**Table 3:** Adjusted mean difference in spine and hip bone mineral density for users and non-users at the 25th, 50th and 75th percentile of BMI

Variables	Adjusted mean difference: spine BMD (g/cm^2^)			Adjusted mean difference: hip BMD (g/cm^2^)		
	Non-users	Users	*p*-value	Non-users	Users	*p*-value
Psychotropics^a^						
BMI 25th percentile	1.196	1.119	0.001	1.039	0.981	<0.001
BMI 50th percentile	1.215	1.162	0.003	1.070	1.032	<0.001
BMI 75th percentile	1.234	1.205	0.121	1.102	1.084	0.106
Antidepressants						
BMI 25th percentile	1.194	1.138	0.024	1.037	0.989	0.001
BMI 50th percentile	1.214	1.174	0.046	1.069	1.045	0.039
BMI 75th percentile	1.234	1.211	0.257	1.010	1.101	0.946
Antipsychotics						
BMI 25th percentile	1.190	1.233	0.746	1.034	1.060	0.732
BMI 50th percentile	1.211	1.226	0.878	1.068	1.072	0.935
BMI 75th percentile	1.232	1.219	0.858	1.101	1.084	0.691
Anticonvulsants						
BMI 25th percentile	1.192	1.072	0.005	1.036	0.958	0.002
BMI 50th percentile	1.213	1.142	0.046	1.069	0.998	0.001
BMI 75th percentile	1.233	1.212	0.617	1.102	1.038	0.007

Note: BMI 25th percentile 24.6 kg/m^2^, 50th percentile 27.1 kg/m^2^, 75th percentile 29.6 kg/m^2^. Final linear regression model is adjusted for age, BMI, smoking, alcohol intake, physical activity level, use of bone positive and negative medications, Index of Relative Socio-economic Disadvantage score, any mood/anxiety disorder and included a BMI × BMD interaction term. BMD, bone mineral density; BMI, body mass index.

a. Antidepressant, antipsychotic and anticonvulsant users are included as psychotropic users.

Similar results were observed for psychotropic use and total hip BMD, where psychotropic use was associated with reduced total hip BMD over the study period (model 1, Table 2). Following adjustments for age and BMI (model 2) and further adjustment for smoking, alcohol intake, physical activity level, use of bone active medications, socioeconomic status and history of mood and anxiety disorders (model 3), the relationship remained constant (Table 2). There was a significant interaction between psychotropic use and BMI in the final model (model 3) (adjusted mean difference −0.256 g/cm^2^, 95% CI −0.392 to −0.119, *p* < 0.001). Psychotropic use was associated with reduced total hip BMD over the study period at the 25th percentile (adjusted mean difference −0.058 g/cm^2^, 95% CI −0.084 to −0.032, *p* < 0.001) and 50th percentile (adjusted mean difference −0.038 g/cm^2^, 95% CI −0.059 to −0.017, *p* < 0.001) of BMI, but no relationship was detected at the 75th percentile (*p* = 0.106; Table 3).

### Antidepressant use

One hundred and fifteen men (12.2%) used antidepressants (SSRIs: *n* = 79, TCAs: *n* = 19, SNRIs: *n* = 12, other antidepressants: *n* = 17) at one or more time points, with a median of 48 months (IQR 13.5−121.5). Antidepressant use was associated with reduced lumbar spine BMD over the study period (model 1, Table 2). Following adjustments for age and BMI (model 2), the relationship remained constant, but this was not the case for model 3 (Table 2). Following adjustments for age and BMI (model 2), antidepressant use was also associated with reduced total hip BMD over the study period (Table 2). In model 3, there was a significant interaction between antidepressant use and BMI at the total hip (adjusted mean difference: −0.292 g/cm^2^, 95% CI −0.438 to −0.146, *p* < 0.001). Antidepressant use was associated with reduced total hip BMD at the 25th percentile of BMI (adjusted mean difference −0.049 g/cm^2^, 95% CI −0.077 to −0.020, *p* = 0.001) and the 50th percentile of BMI (adjusted mean difference: −0.024 g/cm^2^, 95% CI −0.047 to −0.001, *p* = 0.039), but no relationship was detected at the 75th percentile (*p* = 0.946; Table 3) suggesting that the association was stronger among those with the lowest BMI.

Seventy-nine (8.4%) men used an SSRI at one or more time points. SSRI use was associated with reduced lumbar spine BMD over the study period (unadjusted mean difference −0.051 g/cm^2^, 95% CI −0.093 to −0.009, *p* = 0.018). Following adjustments for age and BMI, this relationship remained (adjusted mean difference −0.054 g/cm^2^, 95% CI −0.095 to −0.012, *p* = 0.011). In model 3, SSRI use was not associated with reduced lumbar spine BMD over time (*p* = 0.336). However, there was a significant interaction between SSRI use and BMI in the final model (model 3) (adjusted mean difference −0.404 g/cm^2^, 95% CI −0.707 to −0.102, *p* = 0.009). SSRI use was associated with reduced spine BMD over the study period at the 25th percentile of BMI (adjusted mean difference −0.066 g/cm^2^, 95% CI −0.123 to −0.010, *p* = 0.021), but no relationship was detected at the 50th (*p* = 0.160) or 75th percentile (*p* = 0.927). Similarly, in model 3, there was a significant interaction identified between SSRI use and BMI and total hip BMD (adjusted mean difference −0.392 g/cm^2^, 95% CI −0.572 to −0.213, *p* < 0.001). SSRI use was associated with reduced total hip BMD over the study period at the 25th percentile of BMI (adjusted mean difference −0.058 g/cm^2^, 95% CI −0.091 to −0.025, *p* = 0.001), but no relationship was detected at the 50th (*p* = 0.077) or 75th percentile (*p* = 0.469). Therefore, the association was stronger in men with lower BMI for both lumbar spine and total hip BMD.

No association was detected between SNRI (*n* = 12; 1.3%) or TCA (*n* = 19; 2.0%) use and lumbar spine or total hip BMD. Other antidepressant use (*n* = 18; 1.9%) was associated with reduced lumbar spine BMD (unadjusted mean difference −0.162 g/cm^2^, 95% CI −0.262 to −0.062, *p* = 0.001). Following adjustments, this relationship remained (model 2: adjusted mean difference −0.177 g/cm^2^, 95% CI −0.276 to −0.078, *p* < 0.001; model 3: adjusted mean difference −0.136 g/cm^2^, 95% CI −0.235 to −0.037, *p* = 0.007). There was no association detected between other antidepressants use and changes in total hip BMD.

### Antipsychotics

Over the study period, eight (0.9%) men used antipsychotics (olanzapine: *n* = 5, quetiapine: *n* = 2, risperidone: *n* = 1, paliperidone: *n* = 1), with a median of 46.5 months (IQR 18.3–86.5). No association was detected between antipsychotic use and changes in BMD at the lumbar spine or total hip (Table 2).

### Anticonvulsants

Thirty-two (3.4%) men used anticonvulsants at one or more time points, with a median of 39 months (IQR 5.6–123.3) (pregabalin: *n* = 11, phenytoin: *n* = 9, carbamazepine: *n* = 5, valproate: *n* = 4, clonazepam: *n* = 4, gabapentin: *n* = 2, lamotrigine: *n* = 1, topiramate: *n* = 1, methylcobalamin: *n* = 1). Anticonvulsant use was associated with reduced lumbar spine BMD over the study period (model 1, Table 2). Following adjustments for age and BMI (model 2), this relationship remained constant, but not in model 3 (Table 2). However, in the final model, there was a significant interaction between anticonvulsant use and BMI (adjusted mean difference −0.611 g/cm^2^, 95% CI −1.114 to −0.107, *p* = 0.017). Anticonvulsant use was associated with reduced lumbar spine BMD over the study period at the 25th percentile of BMI (adjusted mean difference −0.120 g/cm^2^, 95% CI −0.205 to −0.036, *p* = 0.005) and the 50th percentile of BMI (adjusted mean difference −0.070 g/cm^2^, 95% CI −0.140 to −0.001, *p* = 0.046), but no relationship was detected at the 75th percentile (*p* = 0.617). Anticonvulsant use was associated with reduced total hip BMD over the study period (model 1, Table 2). Following adjustments for age and BMI (model 2) and further adjustments in model 3, this relationship remained constant (Table 2).

## Discussion

In this longitudinal study of men aged 20 years and over, psychotropic medication use, in particular SSRI and anticonvulsant use, was associated with greater bone loss at the spine and total hip for non-obese men. These relationships persisted after taking into consideration age, socioeconomic status, lifestyle factors and medications known to affect bone.

Our results support the few temporal studies investigating psychotropic medication use and bone loss. SSRI use has been associated with higher rates of bone loss in longitudinal studies of older women^24^ and older men and women.^25^ Among 2722 older women participating in the Study of Osteoporotic Fractures, SSRI but not TCA users had a higher annual rate of total hip bone loss compared with non-users.^24^ Similarly, using data from the Korean National Health Screening Cohort, current and past SSRI use was associated with greater risk of osteoporosis or osteopenia compared to controls irrespective of gender.^25^ These findings are supported by cross-sectional findings showing that SSRI use is associated with lower BMD at the total hip and lumbar spine in older men.^26,27^ In regards to antipsychotic use, decreased bone mass has been previously reported among younger female users,^28^ but not male users,^28,29^ in a number of cross-sectional studies. Similarly, anticonvulsants may also be noxious to bone, but the evidence is limited. In a cross-sectional study of men and women, anticonvulsant use was associated with reduced total hip BMD compared with non-users.^30^ These associations are yet to be explored in longitudinal studies.

Interestingly, BMI was identified as an effect modifier in the association between psychotropic use and bone loss. The negative effects of psychotropic use on bone over time was generally evident for those in the 25th and 50th percentile of BMI, but not at the 75th percentile, suggesting that a higher BMI may be protective against the noxious effects of psychotropics on bone. Interestingly, some studies have reported a positive correlation between BMI and BMD,^31^ with others such as Greco et al^32^ finding that being overweight (BMI 25–29 kg/m^2^) was protective against osteoporosis, whereas obesity (BMI ≥30 kg/m^2^) was associated with an increased risk. Given that a high BMI is associated with metabolic disease and in particular, type 2 diabetes, a link with poor bone health seems valid.^33,34^

The mechanisms underlying the relationship between psychotropic medication use and increased bone loss are not fully understood, with multiple, often overlapping factors likely contributing. Psychotropic use has been shown to directly affect bone turnover, although this effect varies by drug class and individual characteristics.^5^ Psychotropic medications are also associated with an increased risk of falls,^35^ which may further compromise bone health. Additionally, mental disorders themselves are associated with risk factors such as elevated cortisol levels and inflammation, low vitamin D, altered parathyroid hormone and testosterone, all of which are linked to reduced bone mass.^36^ Furthermore, certain lifestyle factors associated with mental disorders, such as smoking, increased alcohol consumption, substance use and a sedentary lifestyle, have been associated with increased risk of bone loss, although these factors did not explain the associations found in this study.

The strengths of this study include (a) the prospective study design and ability to investigate relationships between psychotropic use and changes in BMD over more than a decade of observation, (b) the study population was large and representative of the general population, (c) several potential confounders including mental disorders were taken into consideration and (d) the sample spanned the full adult age range. In regards to limitations, the following should be considered: (a) unidentified confounding needs to be acknowledged; (b) a lack of data on weight-bearing exercise is a limitation, given its influence on bone strength and overall skeletal health; (c) serum levels of vitamin D, parathyroid hormone and testosterone were not available for all participants at each visit; (d) results of this study were obtained for men only, most of whom were White, residing in South-East Australia and thus may not apply to populations of a different ethnicity or to other populations outside the study region; and (e) power restraints affected the investigation of certain psychotropic medication subgroups (i.e. antipsychotics), specific agents and doses.

Over time, even modest reductions in BMD can be clinically meaningful. Absolute BMD changes greater than 0.057 g/cm^2^ at the spine and 0.046 g/cm^2^ at the hip are considered clinically significant,^37^ and such declines are associated with an increased risk of fracture. If psychotropic use is associated with progressive bone loss, regular monitoring and preventive interventions such as calcium and vitamin D supplementation, weight-bearing exercise and pharmacologic therapies should be considered as part of the treatment regimen. In conclusion, our data suggest that psychotropic use is associated with bone loss in non-obese men. This pattern was also observed for users of antidepressants and anticonvulsants. Given that mental health treatments are often lifelong, this information is important in personalised treatment decisions, especially to manage bone health in psychiatric patients.

## Data Availability

The raw data supporting the conclusions of this article will be made available by the authors, without undue reservation.
